# Systems Immunology: Revealing Influenza Immunological Imprint

**DOI:** 10.3390/v13050948

**Published:** 2021-05-20

**Authors:** Adriana Tomic, Andrew J. Pollard, Mark M. Davis

**Affiliations:** 1 Oxford Vaccine Group, University of Oxford, Oxford OX3 7LJ, UK; andrew.pollard@paediatrics.ox.ac.uk; 2 NIHR Oxford Biomedical Research Center, Oxford OX3 7LJ, UK; 3 Institute of Immunity, Transplantation and Infection, School of Medicine, Stanford University, Stanford, CA 94304, USA; 4 Department of Microbiology and Immunology, School of Medicine, Stanford University, Stanford, CA 94304, USA; 5 Howard Hughes Medical Institute, Stanford University, Stanford, CA 94304, USA

**Keywords:** systems immunology, influenza, influenza vaccines, protective immunity, machine learning, data mining, artificial intelligence, multi-omics integrative analysis

## Abstract

Understanding protective influenza immunity and identifying immune correlates of protection poses a major challenge and requires an appreciation of the immune system in all of its complexity. While adaptive immune responses such as neutralizing antibodies and influenza-specific T lymphocytes are contributing to the control of influenza virus, key factors of long-term protection are not well defined. Using systems immunology, an approach that combines experimental and computational methods, we can capture the systems-level state of protective immunity and reveal the essential pathways that are involved. New approaches and technological developments in systems immunology offer an opportunity to examine roles and interrelationships of clinical, biological, and genetic factors in the control of influenza infection and have the potential to lead to novel discoveries about influenza immunity that are essential for the development of more effective vaccines to prevent future pandemics. Here, we review recent developments in systems immunology that help to reveal key factors mediating protective immunity.

## 1. Introduction

Infection caused by the influenza virus has a devastating societal impact, causing up to 650,000 deaths and millions of hospitalizations every year worldwide [1]. High mutation rate, cross-species infectivity and antigenic shift caused by genetic reassortment, i.e., the process in which two different influenza virus strains (human and nonhuman) swap their genes in a common animal reservoir, are causing the emergence of novel influenza strains that have pandemic potential [2,3,4,5]. Especially dangerous are newly emerged avian influenza viruses, such as H5N1 [6] and H7N9 [7] as they cause severe human infection with high mortality rates over 30% for H7N9 [8] and over 50% for H5N1 [9]. The mortality rate of H5N1 strain infection is 20 times higher than that of the 1918 ‘Spanish’ influenza pandemic [3] which caused the worst infectious pandemic in history with over 20 million deaths [10]. The current SARS-CoV-2 pandemic would look trivial in comparison [11,12]. Due to the increased likelihood of emerging avian influenza viruses to cross the species barrier and infect humans, the question is not if but when there will be another pandemic.

Vaccines are the best available measures for the prevention and control of influenza that can reduce the frequency of morbidity, severity of infection and mortality. However, the effectiveness of currently available seasonal influenza vaccines is low compared to other vaccines against viral infections and they often fail to provide protection, especially in the elderly [13]. The reasons for low effectiveness are mismatch with the circulating strains and mutations in vaccine strains caused by the egg adaptation during the vaccine production [14]. Another problem with the currently available vaccines is poor immunogenicity and inability to elicit broader and long-lived immune responses to the same extent as the natural infection, thus vaccine-induced protection depends on annual re-vaccination [4]. In contrast to vaccines, immunity generated by natural infection lasts for decades; indeed, older adults were protected from the 2009 H1N1 pandemic strain by exposure to a structurally similar strain which circulated widely following the 1918 pandemic [15]. The mechanism behind this broad and long-lived immunity following natural infection is unknown but represents a major unmet need to increase the protection (i.e., cross-reactivity) of influenza vaccines and help prevent future pandemics. 

The long-term protection against influenza is multifaceted and complex, requiring the persistence of influenza-specific neutralizing antibodies (‘humoral responses’) and immune effector and memory cells (‘cellular responses’) capable of rapid and effective reactivation upon subsequent pathogen exposure. The best-defined correlate of influenza immunity are antibodies directed against the hemagglutinin (HA), and a hemagglutinin inhibition (HI) titer ≥ 40 is associated with a 50% reduction in risk of influenza infection or illness [16]. However, HI antibody titer provides only a partial explanation of protective influenza immunity in humans [17]. In 1972, Hobson and colleagues, using an influenza human challenge study, identified a group of volunteers with no pre-challenge HI antibody titers, and yet these individuals were protected against influenza infection [18]. Similarly, the protection against pandemic H1N1 strain in 2009 was not dependent on the HI antibody titers [19]. The protection in such cases might be mediated by adaptive immune cells. In contrast to antibodies, T lymphocytes recognize internal antigens which are not under evolutionary pressure and are often more conserved, even in pandemic strains, and therefore could contribute to influenza protective immunity [20]. Indeed, antigen-specific T lymphocytes elicited during primary influenza and parainfluenza virus infections persist at high frequencies for years after viral clearance [21,22]. Moreover, the best defined (and the most immunogenic) human cytotoxic CD8^+^ T cell influenza epitope, M1_58–66_ peptide bound to the HLA-A∗02:01 molecule, is highly conserved within different influenza A subtypes spanning 100+ years [23]. Thus, cytotoxic CD8^+^ T cells might mediate the broad and long-lived immunity and confer protection against illness in individuals lacking humoral immunity to the pandemic strains. While a protective role was shown in mouse and nonhuman primate models [24,25], the role of CD8^+^ T cells in humans remains unknown. Lack of pre-existing helper CD4^+^ T cells has been identified as one of the major predisposing factors for failure to generate antibody response to vaccination [26,27,28,29]. Given the broad potential for cross-protective capacity mediated by T lymphocytes, this aspect of immunity is of considerable interest for the development of novel influenza vaccines that can induce long-lasting protective immunity. Revealing protective influenza immunity represents is a big challenge requiring new concepts that can interrogate immune responses in all their complexity, taking into consideration different arms of immunity. Here, we review how the application of a systems immunology approach—a combination of multi-omics technologies and novel computational methods for integrative analysis—has started to reveal signatures of protective influenza immunity in humans. A better understanding of protective influenza immunity in humans will be essential for the development of a new generation of influenza vaccines that are able to induce long-term immunity against genetically divergent viral strains and prevent future pandemics.

## 2. The Dawn of Systems Immunology—Human Immunology 2.0

Much of the current knowledge about protective influenza immunity comes from animal models, due to the obvious difficulties in researching memory responses in humans. Since the immune system is highly heterogeneous among humans and shaped by many different exposures throughout life, such as exposure to diverse pathogens, environment (food, toxins, allergens, etc.) and commensal microbiota, a larger number of subjects are required than when using animal models to power a study. Furthermore, the exact timing of infection or exposure often cannot be determined precisely, making analysis at specific time points after infection and measurements difficult. Finally, due to difficulties in obtaining and analyzing human tissues samples, most studies have been performed using human peripheral blood samples.

In the past decade, we have witnessed an explosion of new approaches and technologies allowing the exploration of the human immune system [30]. The high-throughput ‘omics’ technologies measuring genes, mRNA (transcriptomics) [31], proteins (proteomics), metabolites (metabolomics), cells (mass cytometry) [32,33] and epigenetic modifications (ATAC-seq) [34] can provide a large amount of data from a very limited number of samples, including blood, secretions or tissue biopsies. In particular, next-generation sequencing has not only facilitated the evaluation of immune cell-specific transcripts and the characterization of T and B cell repertoires [35,36], but also high-throughput single-cell sequencing revealed new information about the heterogeneity of immune cell subsets [37]. Mass cytometry (CyTOF) allows analysis of protein expression at a single-cell level, contributing to the understanding of human immune system heterogeneity and the identification of new immune cell populations [32]. The development of high-throughput platforms, such as ‘systems serology’, is guiding the analysis of the functional properties of antigen-specific antibodies isolated from serum of vaccinated or infected humans [38]. These new approaches and technological developments in systems biology allow examination of the roles and interrelationships of clinical, environmental, biological and genetic factors in the control of infectious diseases [30]. Moreover, by taking advantage of vaccination in combination with multi-omics approaches, ‘systems vaccinology’ has the power to improve vaccine development by identifying key parameters of pre-existing immunological responses associated with protection [30]. 

The basis for protective vaccination stems from a fundamental evolutionary benefit of the host’s immune system to respond more quickly and effectively following re-encounter with pathogens, thus vaccines can be used as tools to deepen our knowledge about immune responses and essential pathways of immunological memory that are associated with protective immunity in humans. The power of the systems vaccinology approach was first demonstrated using the yellow fever live-attenuated vaccine [39,40]. These initial proof-of-concept studies revealed transcriptional signatures involved in antiviral sensing and viral immunity correlating with the magnitude of antigen-specific T cell and neutralizing antibody responses [39,40]. While for many vaccines correlates of protection have not yet been determined, the quantitative correlate of protection for the influenza vaccine is known (HI titers ≥ 40), allowing comparative analysis of the discrete functionalities and subset composition of pre-existing immune cells necessary for establishment of protective immunity. Additionally, since the protection mediated by the seasonal, inactivated influenza vaccine is highly variable across a given population (some individuals do not develop protective Ab titers), this allows the question of which pre-existing immunological parameters are associated with protection (induction of high HI antibody titers) to be addressed. Over the last few years, many systems studies (including ours) used omics technologies to probe the human immune response to influenza vaccination in a comprehensive way and to define new mechanisms and correlates of protective influenza immunity [41,42,43,44,45,46]. The early studies demonstrating the use of systems biology approaches to identify signatures of influenza vaccination revealed that age [44,46], sex [42], pre-existing antibody titers [44], prior vaccination history [47], environmental (rather than genetic) factors [47,48] and post-vaccination immunological parameters (magnitude of plasma blasts and host transcriptional signature after vaccination) [43,45] correlate with protection (mediated by induction of HI antibody titers) [49]. However, the main question on how pre-existing immunological determinants (i.e., immunological memory) can predict the outcome of antibody responses following vaccination has remained unanswered.

## 3. FluPRINT: From Data to Knowledge

Over the past decade, human systems immunology studies have been used to accumulate a wealth of experimental knowledge and technological innovations to monitor and analyze the human immune system more broadly and protective influenza immunity specifically, but often the findings are limited to descriptive studies providing quantitative summaries of biological relationships. It is now time to move towards the next generation of human immune system science, which will allow integrative biological models to be derived, and thereby contribute to more relevant and translational insights into human immunology. So far, most of the approaches using systems biology tools and human studies on influenza vaccination have focused on a single layer of biological data due to the lack of appropriate computational approaches to integrate multi-omics data consisting of many heterogeneous feature types [49,50]. Revealing pre-existing immunological determinants of protective influenza immunity in humans poses a big challenge, requiring new concepts that can combine diverse and high-dimensional data across modalities and clinical studies and model interactions associated with protection [51]. The integration of multi-omics approaches, in particular single cell mass and flow cytometry with transcriptomic profiling, can maximize the informativeness of high-dimensional heterogeneous immunophenotyping [52,53,54,55] to identify determinants of protective influenza immunity, yet the integration and transformation of data into predictive models remains a challenge. Single cell flow and mass cytometry assays vary tremendously in the number of analyzed protein markers, as these technologies have improved considerably over time (number of measured parameters increased from 10 to 40 markers) [56], therefore it is increasingly difficult to compare such data across studies. Nevertheless, these historical data sets have important value as they allow the study of characteristics of larger numbers of individuals across study years and different populations. To resolve this issue, we generated FluPRINT, a unified database for a large-scale study exploring novel cellular and molecular underpinnings of successful immunity to influenza vaccines [57]. There are more than 3000 parameters included in the FluPRINT database measured using CyTOF, phosphorylation-specific and flow cytometry, multiplex ELISA, clinical lab tests, serological and virological profiling. We integrated immunological measurements acquired from 2007 to 2015 from 747 individuals analyzed at the Stanford University Human Immune Monitoring Center. This is a unique source in terms of value and scale that can be used to broaden our understanding of influenza immunity.

## 4. SIMON Says: Humoral and Cellular Immunity Mediate Protection against Influenza

Using the FluPRINT dataset, we are now able to address the question how pre-existing immunity correlates with the outcome of influenza vaccination and begin to model protective influenza immunity in humans. To accomplish that, we developed a novel computational approach—‘Sequential Iterative Modeling Over Night’ (SIMON)—that can integrate heterogeneous, imbalanced, high-dimensional data with a high degree of missing values (i.e., high sparsity) collected across studies and modalities [58,59]. SIMON uses machine learning algorithms to identify patterns and extract knowledge from biomedical data. Machine learning (ML), also known as data mining, a subdiscipline of artificial intelligence, can handle large, heterogeneous datasets even when the expert knowledge is incomplete [60]. ML has just started to be applied to a variety of computational biology problems [61,62] and with profound impact on genomics [63], proteomics (characterization of structure and function of novel proteins [64] and identification of novel protein-ligand interactions, essential for determination of novel T cell antigens [65]), cell image analysis [66], and drug discovery and development [67]. Using SIMON, we have demonstrated that ML can also be used for integration of different data types collected across studies and identification of signatures that correlate with protective immunity after influenza vaccination [59].

In the proof-of-principle study aimed to demonstrate the applicability of ML for integrative analysis, we applied SIMON to a data set collected from 128 donors from five separate clinical studies of seasonal inactivated influenza vaccination, analyzed with various platforms, including single-cell protein expression analysis using CyTOF, to capture both immune system and individual variation available in the FluPRINT database. Using baseline data obtained before the vaccination only, SIMON was able to build high-performance models that could predict if individuals would generate protective influenza immunity mediated by induction of high HI antibody titers one month after vaccination (high vs. low responders). To identify which immunological parameters at baseline are associated with protective immunity (i.e., contribute to separation of high and low responders), in addition to integration and modeling, SIMON performs the feature selection based on the elimination of features and model performance. The identified pre-existing immunological parameters that could predict the outcome of vaccination comprised the frequency of blood-circulating immune cell subsets, including memory B cells and effector-memory CD4^+^ T cells, which had been identified in previous studies [41,44,45], validating the accuracy of our approach. Additionally, SIMON uncovered previously unappreciated immune subsets with an important role in influenza immunity, specifically cytotoxic CD8^+^ T cells (subsets having effector, effector-memory and/or Tc17 phenotype) and CD161-expressing CD4^+^ T cells in mediating protective immunity, revealing that influenza immunity is far more complex than previously thought [59]. Due to their cross-protective potential, identified T cell populations are particularly attractive as targets for the development of broadly protective influenza vaccines. Further assessment of complex signatures identified by SIMON to better reflect the in vivo situation will be essential to guide progress towards the next generation of influenza vaccine capable of inducing broad and long-lived immunity.

## 5. Future Challenges

While SIMON and other machine learning approaches hold promise to identify patterns in the data and reveal the underlying complex interplay of many biological processes, including generation of protective immunity, yet another essential step for building predictive models is preparation of high-quality data. Influenza vaccination is a good proxy for the evaluation of the protective influenza immunity mediated by HI antibodies, but such approaches can fail to identify protection mediated by cellular immunity. One approach to defining correlates of influenza vaccine efficacy is to use the controlled human infection model [68]. In contrast to animal and epidemiological models, human challenge models offer opportunity to investigate human immune responses in a controlled and much more efficient manner [68]. The human challenge model requires fewer participants to power a study, allows investigation of human immunological responses directly, and with the known timing of infection [68]. The human challenge models have been critical for the development of antivirals and vaccines [68]. A few studies that used the human challenge model to investigate immune mechanisms associated with the infection revealed the profound impact of influenza virus on the antibody responses [69] and lymphopenia [70]. Since these studies were performed in the 1970s and 1980s, only a small number of aspects of the immune response were investigated (HI antibodies or lymphocyte counts). However, today, using a systems immunology approach, we can capture the systems-level state of protective immunity, taking into consideration different aspects of the immune system. Moreover, current advances in human in vitro models, such as the recently described human tonsil organoid, can be used to re-capitulate human adaptive influenza responses and could be used to resolve the complexity of the modeling by validation of the key findings [71].

Another important approach to enable training of more detailed models is the integration of biomedical data across clinical studies, just as we did for the generation of the FluPRINT database, but including diverse data from research groups around the globe. The open-data initiatives offer high-quality human immunological data. One example is ImmPort [72], a repository with over 300 clinical studies on allergy, autoimmune diseases, infections (including influenza) and vaccine responses. Integration of open-access data from ImmPort into SIMON will improve the modeling of protective influenza immunity with higher-quality insights. Moreover, the architecture of SIMON software supports both the use of machine learning modeling in the cloud and the server mode, which is essential to accommodate the increased size of data sets, complexity of models and data privacy concerns. The integration of data and sharing of the predictive models between researchers could finally reveal the nature of protective influenza immunity in humans.

## Data Availability

Data sharing is not applicable to this article as no new data were created or analyzed in this study.
